# Co-incubation of Short Amphiphilic Peptides with Dicer Substrate RNAs Results in *β*-Sheet Fibrils for Enhanced Gene Silencing in Cancer Cells

**DOI:** 10.59566/isrnn.2024.0101061

**Published:** 2024-12

**Authors:** Kshitij Gupta, Lorena Parlea, Mathias Viard, Katelyn Smith, Anu Puri, Joseph T. Bergman, Taejin Kim, Bruce A. Shapiro

**Affiliations:** 1 RNA Biology Laboratory, Center for Cancer Research, National Cancer Institute, National Institutes of Health, Frederick, MD, 21702, USA;; 2 Present address: Genes N Life Healthcare Pvt. Ltd., Hyderabad, Telangana,500082, India;; 3 Basic Science Program, Leidos Biomedical, Research Inc., Center for Cancer Research, National Cancer Institute, National Institutes of Health, Frederick, MD, 21702, USA;; 4 Pharmaceutical Sciences, Merck Research Laboratories, Merck & Co., Inc, Kenilworth New Jersey, USA;; 5 Department of Physical Sciences, West Virginia University Institute of Technology, Beckley 25801, West Virginia, USA

**Keywords:** amphiphilic peptides, β-sheet fibrils, RNA interference, Dicer substrate RNA, RNA-peptide co-evolution, peptide folding energy landscape, cancer

## Abstract

RNA can interact with positively charged, amphiphilic peptides to cooperatively assemble into fibrils that enable RNA transport across cancer cellular membranes. RNA decreases the folding energy barrier imposed by the electrostatic repulsion between these charged peptides, thus partaking in RNA-peptide self-assembly along particular pathways in the energy landscape. Specific amphiphilic peptides capable of protecting and transporting RNA across a membrane have Type II’ β-turn hairpin forming motifs in their structures, which aids self-assembly into β-sheet fibrils. We employed a set of such cationic, amphiphilic peptides that have random coiled structures in the absence of folding stimuli, to characterize the (peptides):(RNA) assembly. We subjected these complexes to extensive biophysical characterization *in vitro* and in cell culture. We show that short RNAs (such as Dicer substrate RNAs) can lead these peptides to self-assemble into β-sheet fibrils that have RNA transport capabilities and can act as non-viral delivery vectors for RNA. Modulation in the peptide sequence implicitly alters the way they bind RNA and influence the peptides’ ability to transport nucleic acids across membranes.

## INTRODUCTION

**Fig. 1.**
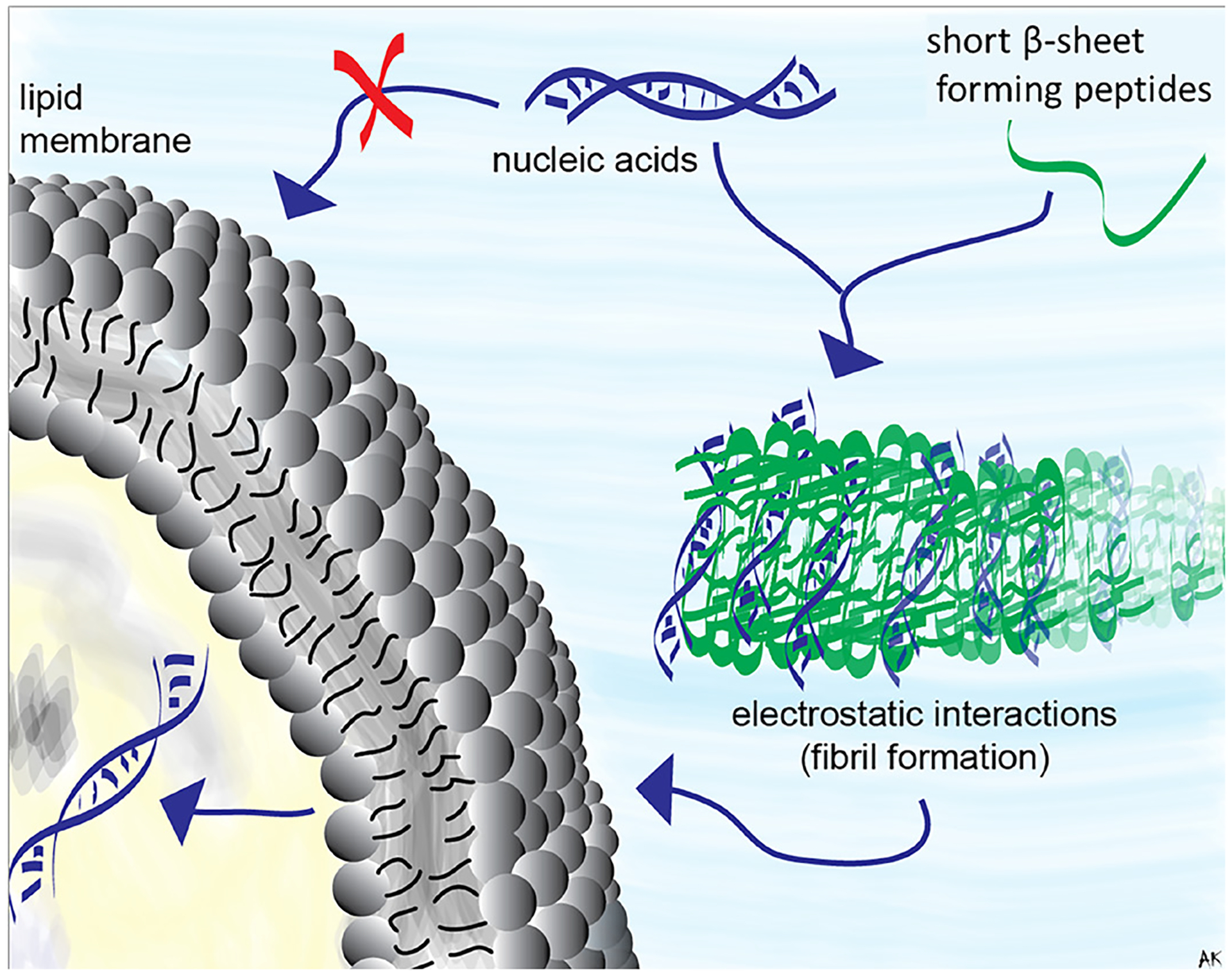
Schematic representation of peptide-assisted transmembrane delivery of nucleic acids.

In recent years, nucleic acid therapies^1^, and in particular RNA Interference (RNAi)-based strategies^2^ have gathered considerable interest in treating various diseases, viral infections, genetic disorders, and cancers. They hold promise for treatment of non-curable diseases^3–5^ and several RNA-based therapies have received approval for clinical use. Yet when delivered alone, unmodified therapeutic nucleic acids encounter biological barriers imposed by their negative charges that prevent cellular entry, nuclease degradation, and lack of target specificity^6–11^. Surmounting these hurdles requires either nucleic acid chemical modification or aided delivery by carriers. Non-viral vectors have demonstrated improved efficacy for the delivery of genes and short therapeutic nucleic acids^3,8,12^. The most common delivery agents used for nucleic acids are polymer- or lipid-based that protect and transport functional nucleic acids across cellular membranes^13,14^. However, many of these carriers suffer from relatively poor uptake, high cytotoxicity, or fail to efficiently release nucleic acids from the endosome, being consumed during endocytic recycling. Recently, short peptides have shown their ability to efficiently deliver nucleic acids into cells both *in vitro* and *in vivo*^15^. These peptides were derived from bacterial^16^ or viral proteins^17^ and are composed of 5–30 cationic and/or amphiphilic amino acids^18^. Nucleic acids can be attached to peptides through covalent conjugation or non-covalent electrostatic complexation^19^. Due to the ease of formulation, here we employ the latter and use rationally designed peptides^20^. Prior to this work, cationic amphiphilic peptides were developed and studied for their antibacterial^21^ and anticancer^22,23^ activities. These peptides form β-sheets in their self-assembled state, with one hydrophilic and one hydrophobic face in response to various stimuli, including electrostatic binding to negatively charged membranes^24–27^. Their sequences are comprised of alternating apolar and polar, positively charged amino acid residues, flanking a specific tetra peptide sequence (-V^D^PPT-) designed to adopt a type IIʹ β-turn^28^. We hypothesized that electrostatic binding to negatively charged nucleic acids will also partake in the self-assembly of β-sheet structures (Fig. 1), and that the resulting complexes would be capable of delivering nucleic acids to cancer cells. As a model RNA sequence, we used functional Dicer substrate RNAs (DsiRNAs) designed to downregulate the expression of enhanced Green Fluorescence Protein (eGFP)^29^, as previously described^30–37^.

Four amphiphilic, β-sheet forming peptides with various sequences (Table 1) were designed to explore the impact of peptide charge, hydrophobicity, and folding propensity. Herein, we explore the formation of (peptide):(RNA) complexes including conformational changes of the peptides in the presence of RNA, relative binding affinities, nucleic acid protection against nuclease degradation, cellular uptake, and gene silencing efficiencies. We demonstrate that co-incubation of peptides with nucleic acids leads to fibrils that are morphologically different from those fibrils formed by peptides alone. These complexes engage in energy-dependent endocytosis and energy-independent pathways during cellular uptake. This interdependency between peptides and nucleic acids to form functional complexes suggests the possibility of a co-evolution and symbiotic relationship between RNA and peptides that might have existed at the early stages of cellular evolution.

## RESULTS

### Peptide design and selection rationale

Amphiphilic peptides, known to act as transporters of various cargoes across cellular membranes^38,39^, were chosen as a model to study our hypothesis that in some cases nucleic acids can partake in self-assembly and β-sheet fibril formation, and that such complexes can deliver functional RNAs intracellularly. Four such amphiphilic peptides were selected based on their amino acid properties, i.e. the total number of positive charges, the hydrophobic vs. hydrophilic content, and their β-sheet assembling capacity, with the expectation that these parameters influence their ability to bind and release RNA (Table 1). The peptide:RNA complexes were characterized by biochemical means. Each peptide is comprised of 20 amino acids with alternating hydrophilic and hydrophobic residues flanking a 4-amino acid type IIʹ β-turn promoting sequence (-V^D^PPT-). These peptides vary in their propensity to undergo stimuli driven assembly with high pH, salt, temperature or negatively charged membranes and adopt β-sheet structures in their fibrillar state^21,25,28,40,41^.

Among the four peptides considered in this study, MAX35^40^, contains 8 polar basic lysine residues alternating with four hydrophobic valine residues in positions 3, 7, 16 and 20, and four isoleucine residues in positions 1, 5, 14, and 18. At physiological pH (7.4), MAX35 presents a +9 positive charge due to the side chain protonation of 8 lysine residues (pKa ~10.53) and protonation of the terminal amino group of the peptide chain. Its protonated lysine residues bind to the negatively charged lipid membranes and induce a transition from a random structure to β-sheets^40^. In addition, MAX35, due to the presence of four isoleucine residues, displays increased hydrophobicity compared to the other peptides considered. It is also capable of stimuli driven self-assembly through lateral and facial association into elongated, well-ordered, β-sheet fibrillar structures^42^. However, MAX8V16E (used as a control) has two negatively charged polar glutamic acid residues at positions 15 and 16 that should render this peptide incapable of adopting a β-sheet conformation.

Two additional amphiphilic peptides are introduced here to study the impact of peptide charge and guanidinium groups of arginines vs. primary amine of lysines: HPL24, where two lysine residues were substituted with neutral but polar glutamines in positions 4 and 15, has a lower total charge (+7) compared to MAX35 (+9); and an HPL24 analogue, MAXR6Q2, where four lysines were replaced with highly basic arginines at positions 6, 8, 13, and 17. Like MAX35, HPL24 and MAXR6Q2 peptides follow similar design principles and thus it can be assumed that in the presence of similar stimuli these peptides can also assemble into elongated, well-ordered fibrillar structures, forming β-sheets in their fibrillar state.

Since negative membranes interact with these peptides, we hypothesized that negatively charged nucleic acids can also electrostatically interact with these positive peptides and form β-sheet fibrils (except MAX8V16E) and the resulting complexes would be able to protect and transport nucleic acids across the cellular membranes. The arginine’s guanidinium group confers an increased electrostatic strength to form multiple interactions with negatively charged molecules compared to that of lysine’s primary amine^43–45^. Thus, MAXR6Q2 is hypothesized to show improved binding to negatively charged interfaces including membrane lipids and nucleic acids, yet might display a decreased nucleic acid release in comparison to HPL24.

### Circular dichroism (CD) studies

**Fig. 2.**
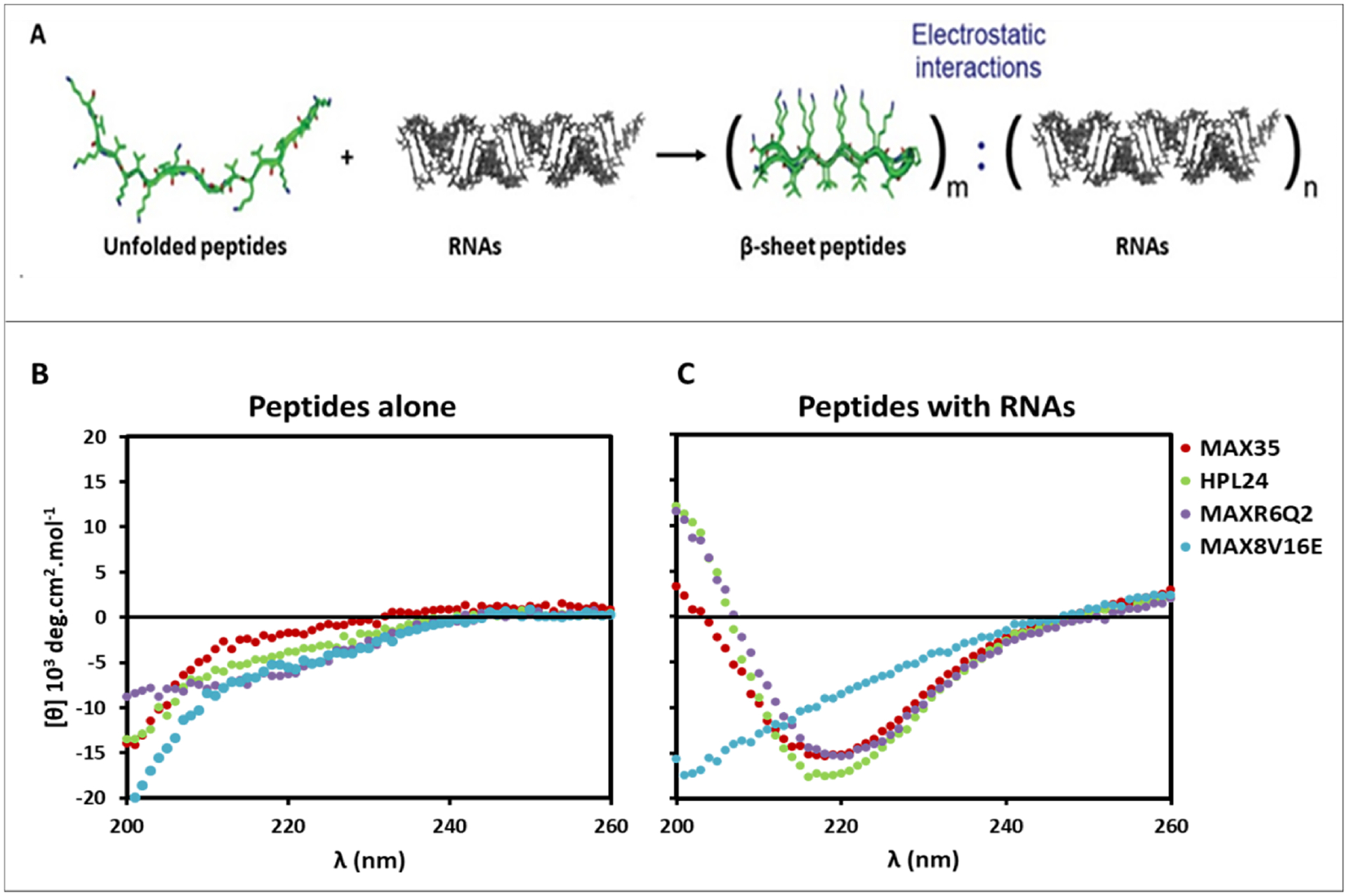
(Peptides):(DsiRNA) complexes. (A) Schematic representation of (peptide):(RNA) complexes. m and n are the number of molecules of peptides and nucleic acids. Various m:n ratios were tested. (B-C) CD studies of peptides at room temperature. (B) Peptides in water without RNA. (C) Peptides co-incubated with DsiRNA were assembled in assembly buffer, pH=8.3 and then diluted in water to final pH of 7.2–7.4 (shown here). All peptides except MAX8V16E form β-sheets in the presence of DsiRNAs.

MAX35 assembles and adopts β-sheet structures in the presence of negatively charged liposomes^40^. We expect the derived peptides, HPL24 and MAXR6Q2, will have a similar behavior. In addition, we extrapolated that these peptides would also adopt a β-sheet conformation in their fibrillar state when interacting with negatively charged nucleic acids (Fig. 2A). To test this, CD experiments of peptides with DsiRNA were performed. The results confirmed that the peptides (except the non-folding, control peptide MAX8V16E) adopt a β-sheet rich structure when co-incubated with negatively charged nucleic acids, as indicated by the minima of mean residue ellipticity at 218 nm (Fig. 2C). The peptides in water, e.g. without external stimuli, did not display any β-sheet formation (Fig. 2B).

### Transmission electron microscopy (TEM)

**Fig. 3.**
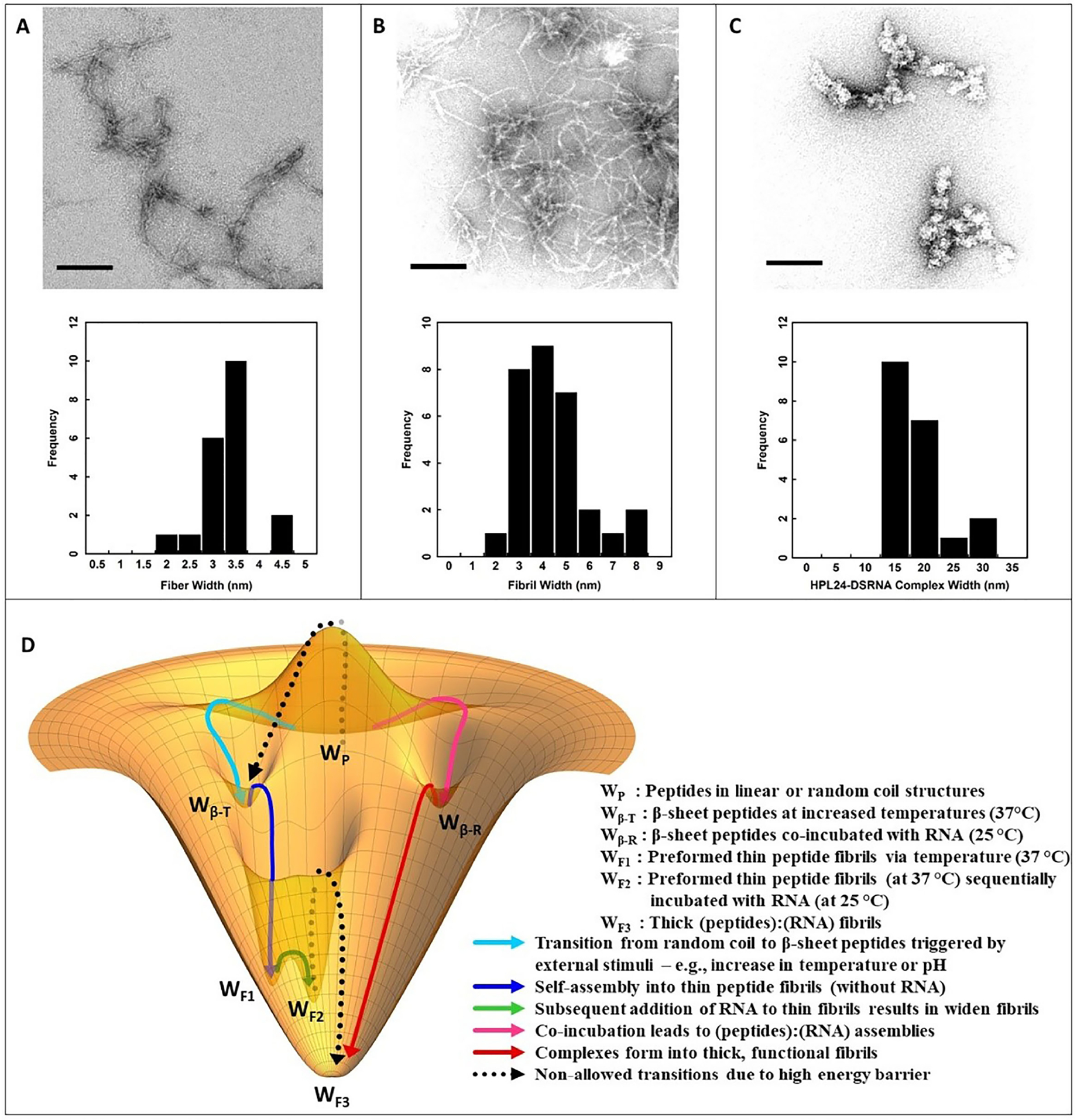
Characterization of (peptide):(DsiRNA) complexes *in vitro*. (A-C) TEM images and data analyses. (A) Preformed fibers of HPL24 peptides alone; (B) Adding DsiRNAs to preformed HPL24 fibers displays slightly thicker fibril formation upon complexation; (C) HPL24 peptides co-incubated with DsiRNA shows big structural changes with significant thickening of the fibrils; Scale bar corresponds to 200 nm. (D) Proposed energy landscape for β-sheet forming peptides (see Discussion for details).

The structures formed by these peptides in the absence and in the presence of nucleic acids were visualized by TEM. The samples for TEM were prepared according to established protocol^46^. Fig. 3 shows representative images for such assemblies of HPL24 as we expect similar results for the other amphiphilic peptides. HPL24 alone (Fig. 3A) formed fibrils when that are 3–3.5 nm in width. Addition of DsiRNAs to preformed fibrils had little effect on the size of the complexes (Fig. 3B), while HPL24 co-incubated with DsiRNA formed thick bundles 15–20 nm in diameter (Fig. 3C). The width suggests that multiple (peptide):(RNA) complexes (approximately 4 – 5) are laminated together to form fibril-like structures. This indicates that short nucleic acids interact with the peptides and form β-sheet fibrils. Fig. 3D depicts a putative energy landscape for (peptide):(RNA) interactions (see Discussion for more details).

### Fluorescence anisotropy (FA) studies

**Fig. 4.**
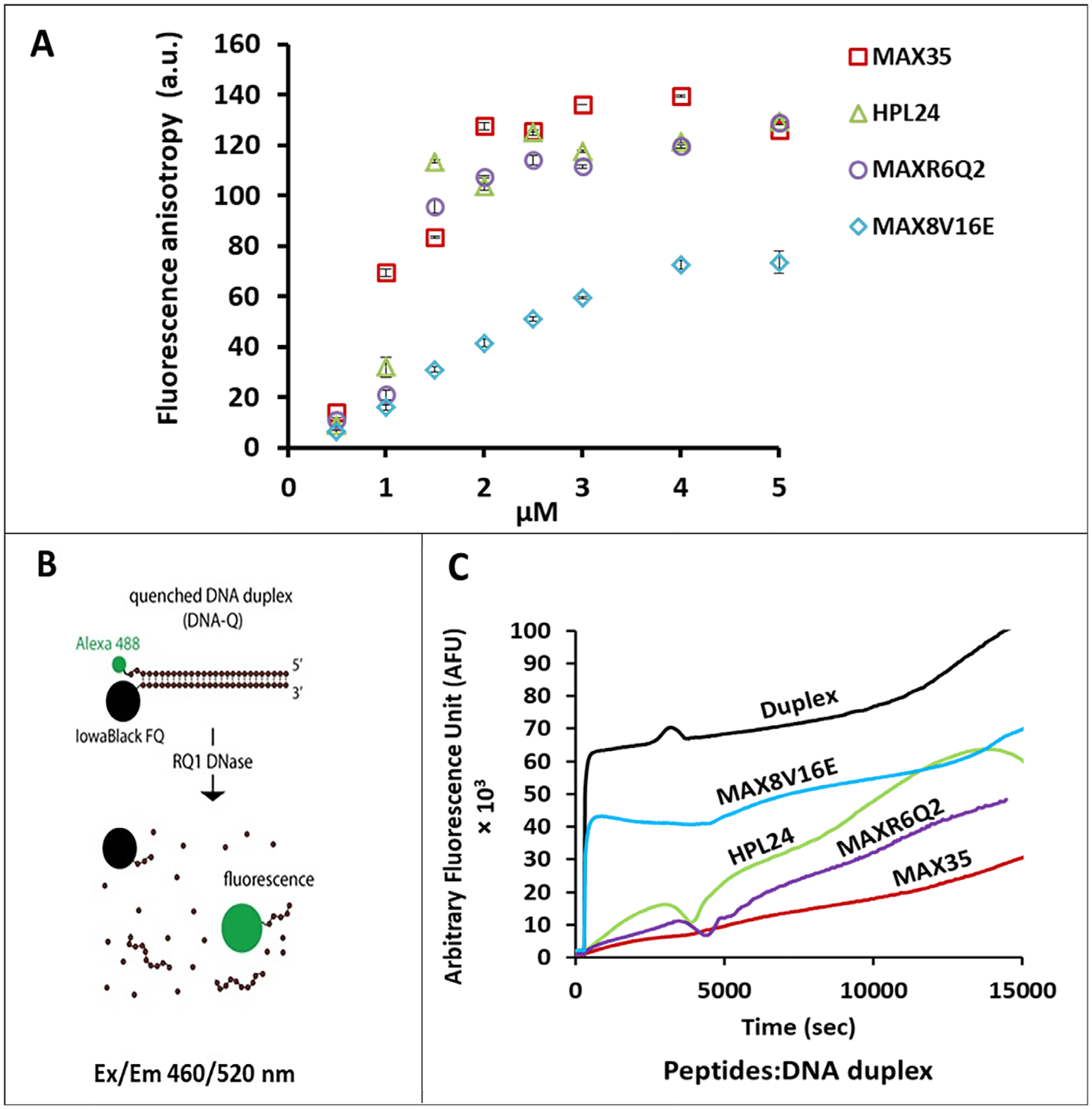
(A) Relative binding affinities assessed with FA experiments. All the peptides show various degrees of binding with Alexa488 labeled DNA duplexes, MAX8V16E shows significantly less. (B) Principle of nuclease degradation. (C). Nuclease degradation assay of fluorescently quenched DNA duplexes alone or complexed with peptides. MAX8V16E showed the least protection.

The (peptide):(nucleic acid) binding affinities were determined by FA in the presence of fixed amounts of Alexa488 labeled nucleic acids (Fig. 4A). The FA values depend on the tumbling of the fluorescent molecules. The slower tumbling (when hindered by binding) corresponds to increased FA values. Fluorescently labeled nucleic acids (100 nM) were co-incubated with different concentrations of amphiphilic peptides (0.5 – 5 μM). With increased concentrations of peptides, higher FA values with respect to the control were observed, suggesting a dose dependent binding of peptides to nucleic acids. Results for (peptide):(DNA) complexes are shown in (Fig. 4A). MAX8V16E also showed interactions with the anionic duplexes; however, the values were significantly lower than that of the fibril forming peptides. This suggests that MAX8V16E did not bind as effectively to the nucleic acids as the other sequences. More importantly, the inability of MAX8V16E to assemble into β-sheet fibrils results in the decrease of local cationic charge density in the milieu of complexes, thus potentially reducing the likelihood of this sequence electrostatically binding RNA.

### Nuclease degradation assay

The ability of peptides to protect nucleic acids against nuclease degradation was assessed as a function of the degree of protection. Fluorescently quenched DNA duplexes (50 nM) complexed with co-incubated peptides (1.5 μM) were subjected to DNase treatment^47^. The concentrations used were based on the results obtained from the FA studies (see above) and cell culture experiments (see below). The fluorescence increase, as a result of dequenching during DNase digestion, was the measure of the degree of nuclease degradation (schematics in Fig. 4B). Thus, unprotected DNA duplexes undergo rapid degradation, while duplexes associated with peptide fibrils degraded slowly (> 4h). Conversely, the non-folding sequence MAX8V16E was unable to protect duplexes and showed rapid degradation soon after DNase addition, supporting the idea that formation of peptide fibrils leads to stronger interactions with nucleic acids (Fig. 4C).

### Transfection efficiencies

**Fig. 5.**
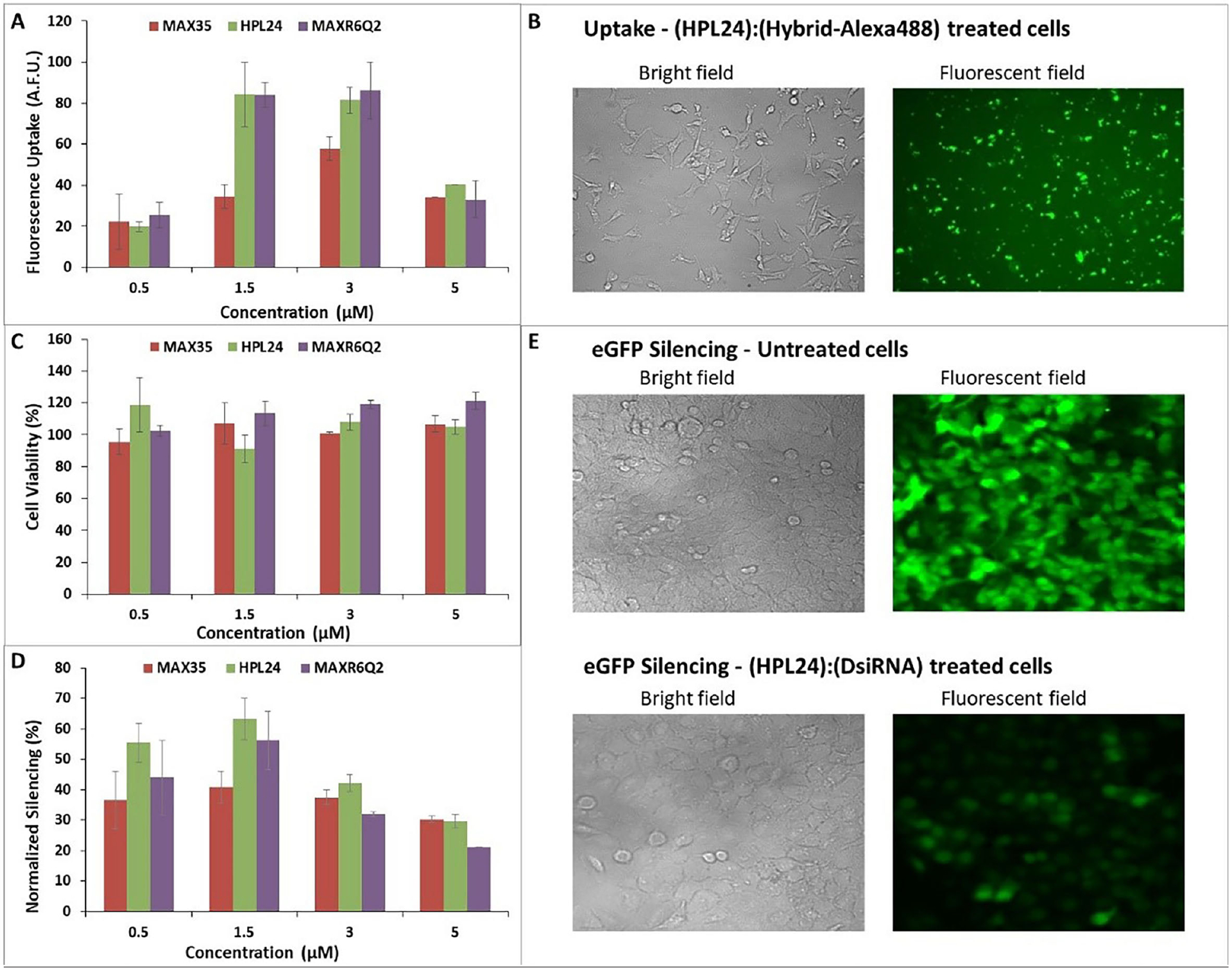
Cellular studies of amphiphilic peptides complexed with DNA/RNA hybrids or DsiRNAs in the breast cancer cell line MDA-MB-231. (A) Relative cellular uptake of fluorescently labeled hybrids complexed with the studied peptides. (B) Visualization of cellular uptake of (HPL24):(hybrid labeled with Alexa488) complexes. (C) (Peptide):(DsiRNA) complexes’ cytotoxicities (D) Percentage of eGFP silencing by DsiRNA mediated by amphiphilic peptides. The eGFP expression was analyzed with flow cytometry and normalized to untreated cells. (E) Visualization of eGFP silencing in MDA-MB-231/eGFP by (HPL24):(DsiRNA) complexes.

Cellular uptake was estimated as the ability of (peptide):(nucleic acid) complexes to bind to as well as be transported into human breast cancer cells (MDA-MB-231). RNA:DNA hybrids were assembled from the RNA sense strand and a complementary DNA antisense strand fluorescently tagged with the Alexa488 fluorophore at the 3ʹ end. To monitor the uptake, peptides at various concentrations (0.5–5 μM) were complexed with the fluorescent hybrids (100 nM) and transfected into cells. 24h post-transfection, cells were washed, and the cellular uptake was analyzed by flow cytometry and fluorescent microscopy (Fig. 5A and B respectively). Peptides capable of forming β-sheet rich fibrils through co-incubation with the nucleic acids efficiently delivered the fluorescent hybrid duplexes. MAX8V16E, which does not form a β-sheet structure, did not show any significant uptake (Fig. S1A), supporting again our hypothesis of a cooperative relation between the peptides and RNA during cellular uptake. As predicted, HPL24 and MAXR6Q2 showed higher uptake overall in comparison to the MAX35 peptide. This higher uptake was observed at 1.5 and 3 μM peptide concentrations.

### Cell viability assay

The conversion of resazurin (blue) to resorufin (pink) of Cell Titer Blue reagent by cells was used to measure cell viability of the MDA-MB-231 breast cancer cells treated with co-incubated (peptide):(DsiRNA) complexes^48^. The samples at different concentrations were added in triplicate to the cells. None of the (peptide 0.5–5 μM):(DsiRNA 100 nM) complexes showed any impairment in the viability of the breast cancer cells (Fig. 5C).

### Gene silencing efficiency

The ability of fibrils to protect RNA and transport it across membranes, while conserving RNA function, was studied through gene knockdown assays. For this purpose, the degree of eGFP silencing was chosen as a measure of RNA function preservation, i.e. the silencing efficiency of DsiRNAs released from the (peptide):(nucleic acid) complexes. DsiRNAs (100 nM) were co-incubated with peptides at different final concentrations (0.5–5 μM) and transfected into human breast cancer cells stably expressing eGFP (MDA-MB-231/eGFP). Three days after transfection, eGFP knockdown, measured as the decrease in the fluorescence signal, was evaluated with flow cytometry and fluorescence microscopy techniques. Three of the peptides preserve the RNA’s function (Fig. 5D). MAX8V16E-delivered DsiRNAs, the peptide that does not form proper β-sheet fibrils did not show any significant silencing effect, as expected (Fig. S1B). Fig. 5E shows representative fluorescence microscopy images of untreated cells and eGFP silencing by (HPL24):(DsiRNA) complexes. Peptides alone and DsiRNAs alone were not able to show any silencing of eGFP expression (data not shown).

**Fig. 6.**
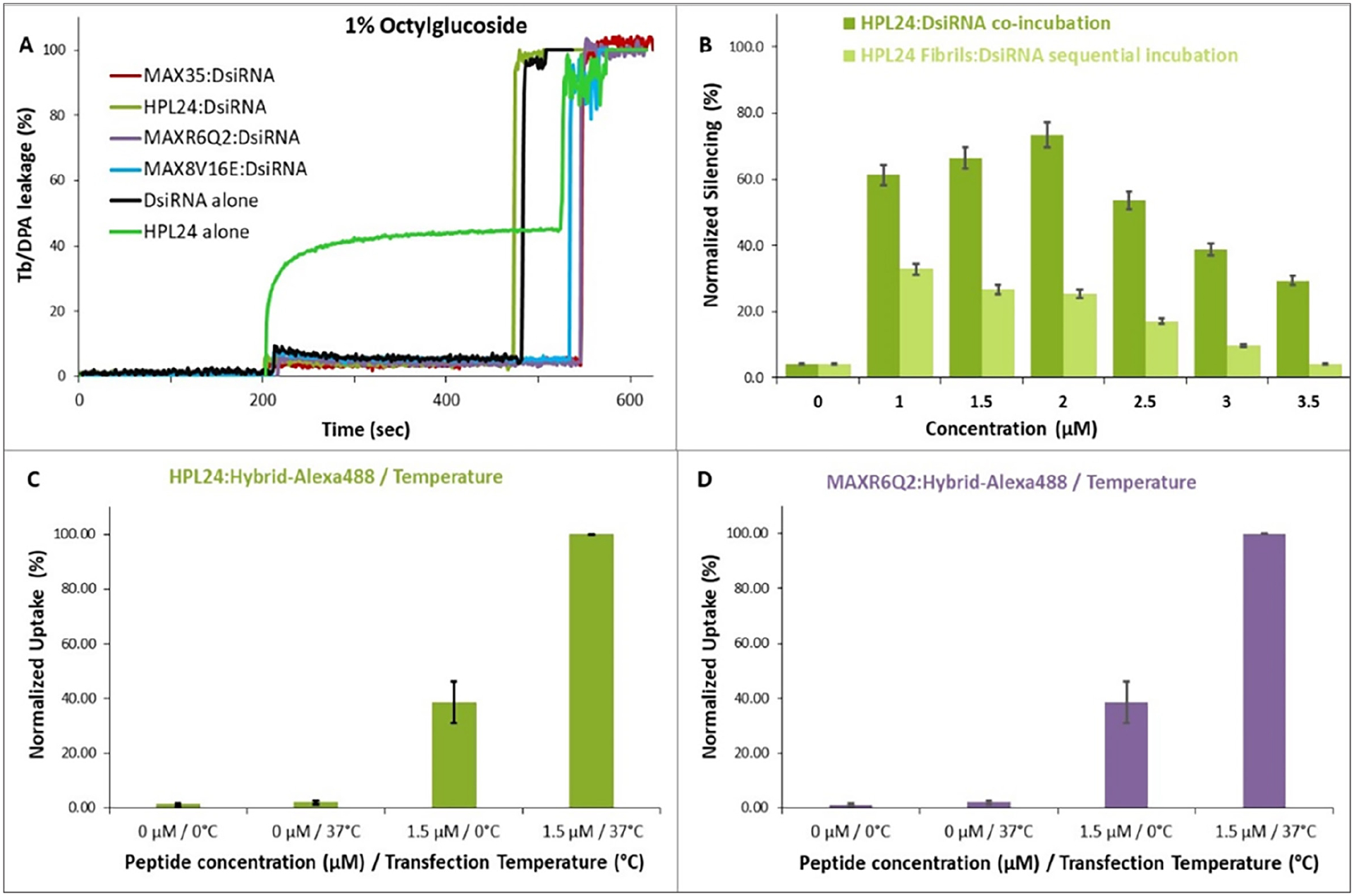
Characterization of (peptide):(nucleic acid) complexes entry mechanisms (A) Tb^3+^:DPA leakage is induced by HPL24 peptide alone but not by the co-incubated (peptides):(DsiRNA) complexes. (B) eGFP silencing in MDA-MB-231/eGFP with DsiRNAs co-incubated with HPL24 or sequentially incubated with HPL24 preformed fibrils. (C-D) Temperature dependent uptake at 0°C and 37°C in MDA-MB-231 cells of hybrids labeled with Alexa488 mediated by HPL24 (C) and MAXR6Q2 (D).

When fibrils were preconditioned to assemble in the absence of DsiRNAs, the complexation of the preformed fibrils with DsiRNAs, (fibrils 1.5 μM):(DsiRNA 100 nM), did not result in significant changes in their structure (Fig. 3B). Likewise, preformed fibrils followed by the addition of DsiRNAs were not able to silence eGFP efficiently (Fig. 6B), implying that peptides need co-incubation with RNA in order to form functional complexes.

### Liposome leakage studies

To evaluate the entry mechanism and whether (peptide):(nucleic acid) complexes lead to pore formation or gain entry through energy-dependent, endocytic means in cancer cells, liposome leakage studies were performed. POPC:POPS (1:1) liposomes with 2% PEG-PE (to avoid liposome aggregation) were chosen as a membrane model, since they mimic the negatively charged outer membrane composition of cancer cells^22,49^. The amphiphilic peptide MAX35 causes Tb^3+^:DPA leakage and forms barrel stave pores in the POPC:POPS:PEG-PE (1:1:0.02) liposomes, as shown previously^40^. Interestingly, none of the co-incubated peptide (1.5 μM) with DsiRNA (100 nM) complexes showed Tb^3+^:DPA leakage from POPC:POPS liposomes. This contrasted with the control experiment, which showed that when the HPL24 peptide was incubated alone with liposomes, liposome leakage was induced, the same as with MAX35 alone (Fig. 6A). It appears that when the peptides associate with nucleic acids there is not a sufficient amount of free peptides available to cause membrane permeation.

### Temperature dependent uptake

To determine whether the uptake of nucleic acids mediated by the fibril forming peptides uses energy-dependent processes, such as endocytosis, comparative studies were performed at the physiological temperature of 37°C and at the low temperature of 0°C, the temperature where the endocytic processes are arrested. Alexa488 labeled RNA:DNA hybrids (100 nM) co-incubated and delivered by 1.5 μM HPL24 or MAXR6Q2 displayed different uptakes at 0°C and 37°C (Fig. 6C and 6D). The data presented were normalized to 100% uptake of nucleic acids at 37°C. At 0°C, the uptake was ~60% lower than the uptake observed at 37°C. Yet, since it could not be completely repressed and approximately ~40% uptake was recorded at 0°C, only part of the uptake might be due to endocytosis. Various mechanisms might be responsible for such (peptide):(RNA) complexes entry into cancer cells, including energy-dependent and energy-independent pathways.

## DISCUSSION

The ability of RNA to interact with cationic, amphiphilic peptides and assemble into β-sheet fibrils was monitored through various biophysical assays, including CD and TEM studies. The degree of RNA protection, transport across membranes, intracellular release and function preservation was monitored through various fluorescent and RNAi assays. RNAi is a process that downregulates targeted gene expression by interfering in message translation^50,51^. We used DsiRNAs designed to silence eGFP^29^ that have been successfully used in our laboratory for gene knockdown both *in vitro* and *in vivo*^31,33–35^.

Initially, we confirmed through CD studies that the DsiRNAs interact with the peptides, to assemble and fold into β-sheets, except MAX8V16E which does not. Additionally, TEM visualization confirmed the predisposition of these peptides to form higher order fibrillar structures, although different size peptide complexes were formed depending on the presence or absence of the nucleic acids. The HPL24 peptide alone, when triggered, can form fibrils ~3–3.5 nm wide, whereas the peptide co-incubated with DsiRNAs, form thicker fibrils of ~15–20 nm. Notably, when DsiRNAs were sequentially mixed with preformed fibrils only slight structural changes were observed, suggesting that the DsiRNAs could only induce thick fibrillar assemblies through co-incubation, or in other words through synergistic interactions between the RNA and peptides. Furthermore, pre-formed mixed fibrils sequentially complexed with nucleic acids were not capable of effectively transporting and/or releasing RNA intracellularly, as shown by the low silencing efficiencies of the sequentially mixed (preformed fibril):(RNA) complexes.

Thus, we propose a possible peptide folding energy landscape as illustrated in Fig. 3D. Cationic peptides do not spontaneously assemble and fold into β-sheets at room temperature and lower pH, due to the energy barriers posed by inter-molecular electrostatic repulsions between positively charged peptide residues. The energy barrier between W_P_ and W_β-T_ is very significant. Triggering mechanisms such as increase in pH, salt concentration or temperature can assist in fibril formation^52^. The pH increase reduces electrostatic repulsion by deprotonating some of the positively charged lysines. Increased salt concentration also diminishes electrostatic repulsion by shielding positively charged residues. Increased temperature reinforces the hydrophobicity and minimizes the entropic penalty of the solvation shell so that peptides assemble and fold into β-sheets via enhanced hydrophobicity (W_P_ → W_β-T_ → W_F1_ in Fig. 3D). When RNA is appended subsequent to fibril formation, the RNA is not capable of disrupting the hydrophobicity and hydrogen bond network within the preformed fibrils, and hence cannot properly intercalate between peptides. Thus, RNA may only cover the surface of the fibrils, as indicated by the slight increase in fibril thickness (Fig. 3B and W_F1_→W_F2_ in Fig. 3D). As such, the (preformed fibrils):(RNA) complexes do not show efficient cellular transfection or gene silencing.

However, when peptides and RNAs are co-incubated, RNA lowers the energy barrier of electrostatic repulsion between the peptides (Fig. 3D W_P_ → W_β-R_). Thus, the peptides assemble and fold into β-sheets producing thicker fibrils, forming optimal (peptide):(nucleic acid) complexes suitable for the RNA protection and transport across membranes. These complexes seemingly form multilayer (peptide):(RNA) fibrils (W_β-R_→W_F3_ in Fig. 3D). Uptake of the nucleic acids by the cells indicates the formation of stable (peptide):(RNA) complexes that bind to cellular membranes and enter into the cells. In addition, effective gene silencing implies translocation of the complexes across the membrane and RNA protection, delivery, entry, and function preservation, as well as intracellular release of DsiRNAs. However, in the case of MAX8V16E, it is possible that the two negatively charged polar glutamic acid residues impede the proper association of these peptides with nucleic acids, in addition to the lack of β-sheet formation. The transfection results corroborate this, MAX8V16E (peptide):(nucleic acid) complexes do not show significant uptake or silencing (Fig. S1A and S1B).

Overall, this work supports the hypothesis that these peptides can be used as nucleic acid carriers, as well as the need for an inter-dependent relationship for successful RNA transport across membranes. Our studies validated the rationale of the peptide selection process. The chosen peptides displayed transfection of nucleic acids and release of DsiRNAs for function, i.e. silencing eGFP. Dose dependent uptake efficiency was investigated for Alexa488 labeled RNA:DNA hybrids by peptides in human breast cancer cells (Fig. 5A). Fibril forming peptides HPL24 and MAXR6Q2 that contain polar uncharged glutamines displayed the highest uptake (~81–86%) when compared to MAX35, which contains lysines (~58%). The uptake is a combined measure of the binding of fluorescent nucleic acids to the cell membranes as well as the amount being transported inside the cells. Uptake efficiency of these peptides was also compared with the widely used commercial transfection agent Lipofectamine 2000 (L2K) (Fig. S1A). All these amphiphilic peptides displayed significantly higher uptake of hybrids than L2K. Peptides HPL24 and MAXR6Q2, exhibited 71% and 73% more uptake respectively than L2K.

Release of DsiRNAs from the (peptide):(nucleic acid) complexes in the cytoplasm is required for gene silencing via the RNAi machinery. It is interesting to note that although the uptake of HPL24 and MAXR6Q2 at 1.5 μM and 3 μM is quite similar, the eGFP gene silencing efficiency is highest at 1.5 μM (Fig. 5D). At higher peptide concentrations the silencing effect is lower. This could be attributed to a reduction in the release of DsiRNAs from the peptides at 3 μM and 5 μM due to relative sequestering by the positively charged peptides given the same concentration (100 nM) of DsiRNAs. Of note, however, is that at the lowest peptide concentration of 0.5 μM an almost comparable hybrid uptake efficiency was observed for all the amphiphilic peptides. Nevertheless, at this concentration, the degree of silencing by DsiRNAs delivered by HPL24 or MAXR6Q2 was higher than MAX35, reinforcing the role of the lower number of positive charges in HPL24 and MAXR6Q2 relative to MAX35 (+7 vs. +9 respectively) in releasing the DsiRNAs. MAXR6Q2, which due to the arginines with guanidinium groups in place of the lysines at certain positions compared to HPL24, can form multiple interactions with the nucleic acids and the cell membrane, thus facilitating higher uptake of the nucleic acids. However, as we expected, it does not seem to display better silencing compared to HPL24. This is most likely due to the additional interactions of the guanidinium groups with the nucleic acids compared to lysine’s primary amine, which leads to reduced release of nucleic acids. At the optimal concentration of 1.5 μM, HPL24 and MAXR6Q2 present better nucleic acid delivery and higher eGFP silencing. It can be inferred from these results that optimizing the number of positive charges, the hydrophobicity, and the (peptide):(nucleic acid) ratio is key to eliciting greater delivery, efficiency, and functionality. As expected, the control, non-β-sheet forming peptide MAX8V16E did not show any significant uptake or eGFP silencing. Our results confirm that not any peptide can assemble into an efficient transporter, and the relation between RNA and peptide is interdependent and symbiotic. Thus, we demonstrated that these fibril forming peptides transport nucleic acids across cellular membranes and release their cargo intracellularly, without loss in RNA’s function, corroborating our hypothesis.

It was previously demonstrated that such amphiphilic peptides could induce leakage in these cancer cells-mimicking liposomes^40^ through pore formation, a physical process exploited by certain molecules for cellular entry (data presented here for HPL24). Since (peptides):(nucleic acid) complexes do not induce liposome leakage, other modes of entry into negatively charged cell membranes have to be exploited. Thus, (peptide):(nucleic acid) complexes might use endocytic processes for cellular entry. Such evidence for an endocytic entry process was also inferred from temperature dependent uptake studies. At 0°C, the endocytic processes should be arrested; however, the uptake efficiency was only ~60% lower than at 37°C. Therefore, lowering the temperature did not suppress the uptake completely. This indicates that energy-dependent endocytic processes are responsible only in part for (peptide):(RNA) transport inside the cells. Furthermore, preformed fibrils complexed sequentially with nucleic acids may be taken up by the cells and release the DsiRNA, (Fig. 6B), yet these complexes were not able to silence eGFP efficiently. This suggests that an interdependent relationship and co-incubation between peptides and RNA is required for formation of functional complexes. Thus, cellular entry of the functional (peptide):(RNA) complexes is likely due to both energy-dependent and energy-independent pathways, and the latter might be attributed to a residual primordial mechanism.

## MATERIALS AND METHODS

### Materials and Methods are detailed in Supporting Information

#### Syntheses of amphiphilic peptides and β-sheet fibril formation.

The peptides were prepared via Fmoc-based solid-phase peptide chemistry and cleaved from the resin and simultaneously side chain deprotected using a trifluoroacetic acid/thioanisole/ 1,2-ethane-dithiol/anisole (90:5:3:2) cocktail for 2 hours under argon atmosphere. The crude product was precipitated with cold diethyl ether and then lyophilized, followed by purification with reverse-phase HPLC equipped with a semi-preparative Vydac C18 column. The purity of the peptides was verified by analytical HPLC-MS.

Fibrillization of peptides was initiated by adding an equal volume of buffer (100 mM BIS-TRIS propane, 300 mM KF, pH 7.4) to a 300 μM solution of peptide dissolved in ultrapure water. Solutions were mixed via vortexing and heated to 37°C for 30 min to induce fibril formation. Fibrils assemble at high peptide concentrations in ionic strength media and at physiological temperature without forming a gel.

#### Nucleic acid duplex assembly.

The oligos for DsiRNAs targeting eGFP^29^ and the corresponding complementary DNA strands (sequences in Supplementary Information) were ordered from IDT, Coralville IA. The DNA sequences were fluorescently labeled with Alexa488 or Iowa black quencher. The assembly of the corresponding sense and antisense RNA duplexes, DNA duplexes or RNA:DNA hybrids was done as previously described^31^ by denaturing the molecules at 95°C for 2 min followed by annealing at room temperature.

#### Formation of (peptide):(nucleic acid) duplex complexes.

Peptides at different concentrations were introduced to a fixed concentration of nucleic acids solution and incubated for 30 min at room temperature. The resulting complexes were diluted to the desired volume in enzyme-free water, buffer, or serum-free media to get to the desired final concentration required for each particular experiment.

#### Circular dichroism (CD) studies.

CD spectra were collected on an Aviv model 420 Circular Dichroism Spectrometer (AVIV Biomedical, Lakewood, NJ). Wavelength scans were recorded using a 1 nm step size and a 2 second averaging time at 25°C with a 5-minute equilibration time.

#### Transmission electron microscopy (TEM).

Images of (peptide):(RNA) complexes and controls were obtained using a Hitachi H-7650 microscope at 80 kV.

#### Fluorescent anisotropy (FA).

FA measurements were used to determine the binding affinities of peptides to fluorescently labeled nucleic acids using Tecan Infinite M1000 (Tecan, USA).

#### Nuclease degradation assay.

The ability of these peptides to protect nucleic acids against nuclease degradation was assessed as described previously^47^. DNA duplexes complexed with peptides were treated with RQ1 RNase-free DNase (Promega). This enzyme cleaves DNA endonucleolytically, releasing short oligonucleotide fragments, including the Alexa488 labeled end that is quenched in the duplex.

#### Uptake and silencing measurements by flow cytometry.

The uptake of RNA:DNA hybrids and the extent of eGFP silencing were assessed in the human breast cancer (cell line MDA-MB-231) 1- and 3-days post-transfection respectively, by fluorescence-activated cell sorting (FACS) using Cell Quest software.

#### Temperature dependent uptake.

The uptake efficiencies of (peptide):(hybrid) complexes at 37°C and 0°C temperatures in MDA-MB-231 non-eGFP expressing cells were compared using FACS.

#### Fluorescent microscopy.

MDA-MB-231 cells were imaged with a Nikon 200 TE inverted microscope (Melville, NJ) to visualize the uptake or eGFP silencing.

#### Cell viability assay.

The viability of the MDA-MB-231 cells treated with peptides alone or in complexation with DsiRNAs was measured with the Cell Titer Blue assay on a SpectraMax M2 spectrophotometer (San Jose, CA).

#### Tb^3+^:DPA leakage assay.

This assay was performed in a Fluorimeter (Fluoromax-3, Horiba Jobin Yvon, Edison, NJ) equipped with a water bath.

#### Free energy landscape.

The free energy landscape diagram that illustrates the possible peptide folding and fibril formation pathways was created with the software Mathematica (Ver. 11).

## CONCLUSIONS

It was proposed that the co-evolution and coordinated interactions between peptides and RNA was the basis of modern cellular life processes^53–55^. RNA can participate in cationic, amphiphilic peptide assembly into β-fibrillar amyloid (and other higher order) structures. Without environmental factors, these peptides do not simply fold into β-sheets. Assembled (peptide):(RNA) fibrils are efficient protectors and transporters of RNA, as seen by the efficient silencing performed by these complexes. The assemblies are stable in a wide range of conditions, conferring RNA protection in various environmental situations, possibly even in the harsh circumstances of the primitive earth. Our system provides an excellent example that corroborates the co-evolution and co-dependence between RNA and peptides as a proposed concept model for the origin of life. The complexes are able to cross the cell membrane and deliver DsiRNAs without loss of the RNA’s function. Both RNA and peptides are postulated to be instrumental in abiogenesis, as they mutually need each other to achieve modern molecular functions. Thus, we believe that this study brings in an additional piece of proof that a co-evolution and a co-dependent, symbiotic relationship between RNA and peptides might have been employed since the early stages of life.

## Supplementary Material

1

## Figures and Tables

**Table 1.** Sequence and design rationale of the amphiphilic, β-sheet forming peptides used in this study

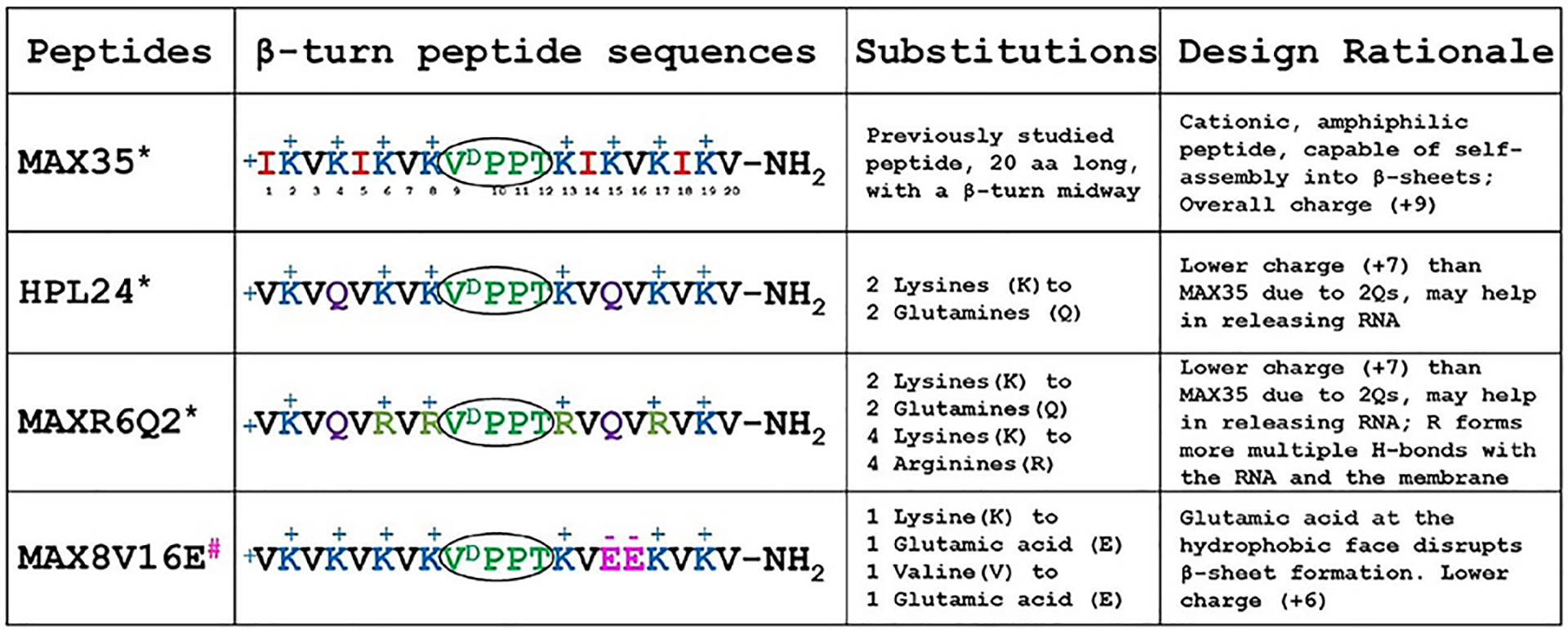

Note:

* β-sheet forming peptides;

^#^ Non-β-sheet forming peptides;

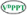
 β-turn promoting sequence
