# Supplementary material for: Co-incubation of Short Amphiphilic Peptides with Dicer Substrate RNAs Results in β-Sheet Fibrils for Enhanced Gene Silencing in Cancer Cells
Source: RNA Nanomed. Author manuscript; Available in PMC 2025 Apr 18. (PMC12007892; doi:10.59566/isrnn.2024.0101061)
Supplement: 1 [file NIHMS2068144-supplement-1.pdf]

SUPPLEMENTARY MATERIAL

Materials and Methods - Detailed Description

Materials. Fmoc-amino acids were purchased from Novabiochem (Billerica, MA). 2-(6-Chloro-1H-benzotriazole-1-yl)-1,1,3,3-tetramethyl-aminium hexafluoro phosphate (HCTU) was purchased from Peptides International (Louisville, KY). All the lipids were purchased from Avanti Polar Lipids (Alabaster, AL). Tb (III) Chloride Hexahydrate ($TbCl_3.6H_2O$), 2,6-Pyridinedicarboxylic acid (Dipicolinic acid, DPA), octyl β-D-glucopyranoside (OG), and other reagents were procured from Sigma-Aldrich (St. Louis, MO). Sephadex G-50 and Sepharose CL-6B were obtained from GE Healthcare Biosciences AB (Uppsala, Sweden). Sense and anti-sense strands for Dicer substrate siRNAs (DsiRNAs), Alexa488 fluorescently labeled RNA:DNA hybrid duplexes, Alexa488 and Iowa black quencher labeled DNA duplexes were purchased from Integrated DNA Technologies, Inc. (Coralville, Iowa, USA). Human breast cancer cell lines MDA-MB-231 and enhanced Green Fluorescent Protein expressing MDA-MB-231/eGFP cells were procured from ATCC (Manassas, VA) and Cell Biolabs Inc. (San Diego, CA) respectively. Nuclease protease free water was purchased from Quality biological Inc. (Gaithersburg, MD), Cell titer blue reagent and RQ1 RNase free DNase were obtained from Promega (Madison, WI). Cell culture reagents and media were purchased from Gibco (Invitrogen, NY).

Syntheses of peptides. Peptides were prepared on PL-Rink Resin in 0.25 mM quantities via automated Fmoc peptide synthesis employing an ABI 433A peptide synthesizer (Applied Biosystems, Snoqualmie, WA) and HCTU activation. The resulting dry resin-bound peptides were cleaved and side-chain-deprotected using a trifluroacetic acid (TFA): thioanisole: ethanedithiol: anisole (90:5:3:2) cocktail. Crude peptides were purified by RP-HPLC (Agilent technologies, Santa Clara, CA) using preparative Grade C18 peptide/protein column (Vydac, Hesperia, CA). Peptides were purified using a linear gradient from solvent A (0.1% TFA in water) to solvent B (90% Acetonitrile, 9.9% water, 0.1% TFA). The purity of peptides was measured by positive mode electrospray ionization–mass spectrometry (ESI-MS, Shimadzu, Columbia, MD). MAX35 ESI-MS: 2287.4 $[(M+H)^+$ calculated 2286.1]; HPL24 ESI-MS: 2230.7 $[(M+H)^+$ calculated 2230.9]; MAXR6Q2 ESI-MS: 2343.4 $[(M+H)^+$ calculated 2342.9]; MAX8V16E ESI-MS: 2262.2 $[(M+H)^+$ calculated 2260.9]. After purification, the peptides were lyophilized, and fresh solutions were prepared prior to each experiment by reconstituting the purified peptides in enzyme-free water. All the peptide sequences used in this study are shown in Table 1 in the main text.

β-sheet fibrils formation. A stock solution of peptide dissolved in ice cold water was prepared, and the concentration confirmed by UV-Vis spectroscopy. The stock solution was then diluted with chilled water to achieve a concentration of 300 µM peptide in a total volume of 200 µL. Fibril formation was initiated by an equal volume of 100 mM BIS-TRIS propane, 300 mM KF (pH=7.4) buffer. This solution was incubated at 37°C for 30 min to allow formation of β-sheet fibrils in physiological temperatures and ionic strength of the media. At this final 150 µM peptide concentration, fibrils form without gelification.

Nucleic acid duplexes assembly. Nucleic acids used in this study were designed to target eGFP[1]: fluorescent and non-fluorescent DsiRNAs, Alexa488 labeled RNA:DNA hybrids, Alexa488 DNA duplexes and quenched DNA duplexes[2,3]. The individual sense and antisense strands of all the designed duplexes, purchased from Integrated DNA Technologies, Inc. (Coralville, IA). The desired duplexes were assembled in the assembly buffer (89 mM Tris-Borate (pH=8.3), containing 2 mM $Mg(OAc)_2$, 50 mM KCl, as described previously, by heating them at 95°C for 2 min followed by annealing at room temperature for 30 min[4].

Nucleic acid sequences used in this project:

• RNA sequences for DsiRNA targeting eGFP[1]:
5′-pAAGGAUGACUAUGUGAAGGCACUGC;
5′-CGGUGGUGCAGAUGAACUUCAGGGUCA.

Fluorescently labeled DNA sequences[3,5,6]:

• DNA-3′ Alexa488:
5′-GGAGACCGTGACCGGTGGTGCAGATGAACTTCAGGGTCAtt/3AlexF488N

• DNA-5′-Iowa Black Quencher (IBQ):
5′–/IBQ/aaTGACCCTGAAGTTCATCTGCACCACCGGTCACGGTCTCC

Formation of (peptide):(nucleic acid duplex) complexes. Fresh stock solutions of peptides dissolved in enzyme-free water were prepared prior to each experiment. The concentrations of all peptides stock solutions were calculated from the recorded absorbance at λ=220 nm, using 15750 $M^{-1}cm^{-1}$ as molar extinction coefficient (ε). Various peptide concentrations were co-incubated with fixed concentration of nucleic acids (DsiRNAs, RNA:DNA hybrids or DNA duplexes) at room temperature for 30 min to obtain the (peptide):(nucleic acid) complexes. These solutions were diluted to the desired volume and final concentration with either enzyme

free water, buffer or serum free media, according to the experiment. In the case of fibril incubation with RNA, the fibrils were also pre-formed, as described above, and subsequently incubated with DsiRNA to obtain the (fibril):(nucleic acid) complexes for TEM and cell culture studies.

Circular dichroism (CD) studies. All the peptides, DsiRNAs, buffers and enzyme-free water were brought to room temperature prior to incubation (~10 min). The peptides were resuspended in water, and the DsiRNA was assembled in assembly buffer, pH=8.3. A 30:1 ratio of (peptides):(DsiRNAs) were co-incubated for 30 min at room temperature and then diluted with enzyme free water to achieve a final peptide concentration of 5 µM (final pH 7.2-7.4) (Fig. 2C). The complexed samples were immediately added to the CD cells. The CD spectra were collected on an Aviv model 420 Circular Dichroism Spectrometer (AVIV Biomedical, Lakewood, NJ). Wavelength scans were recorded using a 1 nm step size and a 2 second averaging time at 25°C with a 5-minute equilibration time. 1 cm quartz cells were used for collecting the spectra. Mean residue ellipticity [θ] was calculated using the following equation: [θ] = (θobs - θblank)/[(10)(l)(C)(r)(1000)] where θobs is the measured ellipticity and θblank is the ellipticity of the assembly buffer without peptides or DsiRNAs, l is the path length of the cell (1 cm), C is the peptide concentration in Molarity (M), and r is the number of residues.

Transmission electron microscopy (TEM). Samples to be imaged by TEM were prepared as in previous studies[7]. Briefly, 1.5 µM peptide with 100 nM DsiRNAs (final concentrations) were incubated at room temperature for 30 minutes. 2 µL of the resulting solution was placed on a 200-mesh carbon coated copper grid. Excess sample liquid was blotted away with filter paper. A solution of 1% uranyl acetate was then added to the grid as a negative stain to enhance contrast between the complexes and the background. Excess stain was blotted away, and the grids were imaged immediately. The images were obtained using a Hitachi H-7650 Transmission Electron Microscope (TEM) at a voltage of 80 kV and analyzed using Image J software.

Fluorescent anisotropy/polarization measurements. Fluorescent anisotropy/ polarization measurements were conducted on a Tecan Infinite M1000 instrument (Tecan, USA) to determine the binding affinities of peptides to fluorescently labeled nucleic acids. Peptides at various concentrations from 0.5-5 µM, were co-incubated with Alexa488 fluorescently labeled RNA:DNA hybrid duplexes, DsiRNAs duplexes, or DNA duplexes (100 nM final concentration). Changes in fluorescent (anisotropy/ polarization) values were used to quantify the binding affinities of nucleic acids to the studied peptides.

Nuclease degradation assay. The ability of these peptides to protect nucleic acid duplexes against environmental factors, such as degradation by nucleases, was assessed by FRET as described elsewhere[8]. In brief, quenched DNA duplexes were used, with the 3′ end of the antisense strand labeled with fluorescent Alexa488 and the 5′ end of the sense strand labeled with fluorescence quencher Iowa Black FQ (Promega, WI). At room temperature quenched DNA duplexes (50 nM, final concentration) alone or complexed with peptides (1.5 µM, final concentration) were incubated for 5 min at 37°C to get stable baseline. The samples were treated with RQ1 RNase-free DNase (Promega, WI), according to the manufacturer's protocol. The degree of duplexes degradation was quantified as a function of the dequenching of Alexa488 at λ_{ex} 460 nm and λ_{em} 520 nm for 4h. The excitation slit width was kept at 2 nm, and the dequenching was recorded every 30 sec.

Cell culture studies. Human breast cancer cells, MDA-MB-231, and the same cell line stably expressing enhanced Green Fluorescent Protein (eGFP) were maintained in a Dulbecco's modified Eagle's medium (DMEM) supplied with 10% (v/v) heat-inactivated FBS (fetal bovine serum), 100 i.u./mL penicillin and 100 µg/ mL streptomycin, under a humidified 5% CO_2 atmosphere at 37°C. The types of cells (expressing or not eGFP) were chosen according to the type of experiment performed: non-eGFP cells for uptake and cell viability, and eGFP-expressing cells for silencing experiments.

Transfection and silencing measurement by flow cytometry. 60,000 cells/well of MDA-MB-231 cells and 30,000 cells/well of MDA-MB-231/eGFP cells in serum containing media were plated on 24 well plates for uptake and silencing experiments respectively one day prior to experiments. On the day of transfection, the media was replaced with serum free media containing different concentrations of peptides complexed with either Alexa488 RNA:DNA hybrid duplexes or Dicer substrate siRNA duplexes (DsiRNAs). The cells were then incubated for 4h at 37°C. After 4h, the serum free media was replaced with serum media. Efficiency of uptake of RNA:DNA hybrid duplexes and the extent of eGFP silencing were statistically analyzed after 1 day and 3 days of transfections respectively by fluorescence-activated cell sorting (FACS) using Cell Quest software.

Fluorescent microscopy. One day post-transfection

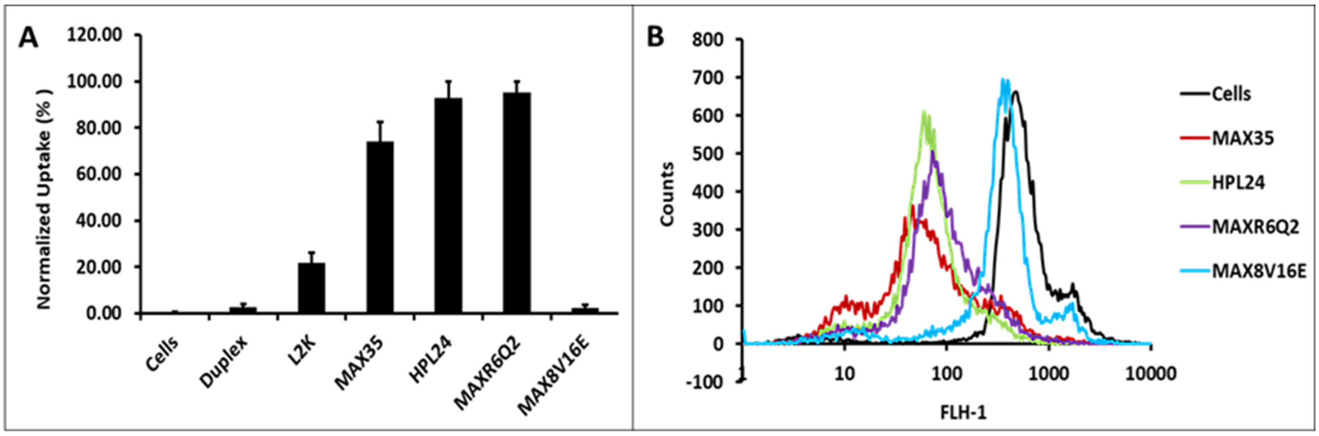

Fig. S1. Cellular studies of amphiphilic peptides at optimal concentration (1.5 μM) complexed with hybrid or Dicer substrate siRNAs (DsiRNAs) in breast cancer cell line MDA-MB-231. (A) Relative cellular uptake of fluorescently labeled duplexes (50 nM) complexed with lipofectamine or the studied peptides (1.5 μM). (B) eGFP silencing by (peptide):(DsiRNA) complexes at 1.5 μM peptide and 100 nM DsiRNA.

(-24 hours) and -three days post-transfection (~72 hours), treated and un-treated MDA-MB-231 and MDA-MB-231/eGFP human breast cancer cells respectively were imaged with a Nikon 200 TE inverted microscope (Melville, NJ) to determine the silencing efficiency of eGFP. For fluorescence imaging, PanFluor 20X, ELWD, NA=0.45 objective, Nikon B-2E/C, 465–495/505/515–555 cube (Chroma Technology Corp., Rockingham, VT), and the Bioprocess software (Universal Imaging Co., Downingtown, PA) were used.

Temperature dependent uptake. 60,000 cells/well of MDA-MB-231 cells were seeded into two separate 24 well plates one day before the transfection. On the day of transfection, both the plates were transfected with HPL24 and MAXR6Q2 (1.5 μM)/alexa488 RNA:DNA hybrids (100 nM) complexes at their final concentrations in serum free medium. One plate was kept at 37°C and another was kept at on ice in a cold room for 4h. After 4h serum free media from both the plates was replaced with serum containing media and both the plates were kept for one more day at 37°C. Next day, the cells were dissociated and analyzed for RNA:DNA hybrid efficiency by FACS to determine the effect of temperature dependent uptake.

Cell viability assay. Viability of the MDA-MB-231 human breast cancer cells were tested on treatment with peptides alone and in complexation with DsiRNAs. Cells were seeded in 96 well plates at a density of 20,000 cells/well in serum containing media 24 hours prior to experiments. Peptides with and without DsiRNAs at various concentrations were added to the cells in triplicate in serum free media and incubated for 4 hours at 37°C. After incubation, the serum free media was replaced with serum containing media for another 24 h at 37°C. At the end of incubation, according to the manufacturer's protocol, cell titer blue reagent was added to each well and the cells were further incubated for 4 hours at 37°C. The fluorescence of the resofurin (converted from resazurin by viable cells) was measured at λ_{ex} 560 nm and λ_{em} 590 nm with an auto cut-off in a fluorescent ELISA plate reader (SpectraMAX, Molecular Devices, Sunnyvale, CA).

Preparation of Tb^{3+}:DPA containing liposomes. 1-palmitoyl-2-oleoyl-sn-glycero-3-phosphocholine (POPC), 1-palmitoyl-2-oleoyl-sn-glycero-3-phospho-L-serine (sodium salt) (POPS), 1,2-distearoyl-sn-glycero-3-phosphoethanolamine-N-[methoxy(polyethyleneglycol)-2000 (PEG-PE) lipids dissolved in $CHCl_3$ were mixed at desired mole ratio 1:1:0.02 in a glass tube and a lipid film was formed by removing $CHCl_3$ under nitrogen at room temperature. Any residual $CHCl_3$ was removed by placing the films overnight in a vacuum desiccator. After hydrating the lipid film using Tb^{3+}:DPA (7.5 mM $TbCl_3$, 75 mM Na citrate, 75 mM Na dipicolinate, 10 mM TES, pH 7.4) solution, samples were mixed by vigorous vortexing to generate multilamellar liposomes. Multilamellar liposomes were sonicated at 4°C by using a Probe Sonicator (Branson Ultrasonics (Shanghai) Co., Ltd, China). Typically, 10 minutes of sonication (1-minute pulses and 1-minute rest) yielded unilamellar liposomes ~100 nm in diameter, as determined by Zeta Nanosizer (Malvern Panalytical, Westborough, MA)). The sonicated liposomes were centrifuged at 1,500 × g for

5–10 minutes to remove any titanium particles and larger aggregates. Un-encapsulated Tb^{3+}:DPA were removed from liposome-entrapped molecules as follows: Tb^{3+}:DPA loaded liposomes were separated from un-entrapped Tb^{3+}:DPA, using a size-exclusion gel chromatography column (Sephadex G-50, 40 cm × 1 cm), pre-equilibrated with buffer 1 (10 mM TES, 100 mM NaCl, 1 mM EDTA, pH 7.4). Liposome-encapsulated Tb^{3+}:DPA was analyzed by measuring fluorescence at λ_{ex} 276 and λ_{em} 545 nm (see below Tb^{3+}:DPA leakage assay).

Tb^{3+}:DPA leakage assay. This assay[9] was performed in a Fluorimeter (Fluoromax-3, Horiba Jobin Yvon, Edison, NJ) equipped with a water bath maintained at 25°C. The samples were placed in a quartz cuvette under constant stirring at a final concentration of 0.1 mM EDTA in buffer 2 (10 mM TES, 100 mM NaCl pH 7.4). The initial fluorescence of Tb^{3+}:DPA (λ_{ex} 276 nm and λ_{em} 545 nm) in the liposomes was set to 100% value. To assess if peptide (1.5 µM):DsiRNA(100 nM) complexes might form pores, and therefore induce membrane leakage, the complexes were mixed with liposomes. The degree of leakage of liposome entrapped Tb^{3+}:DPA was determined by a decrease in fluorescence intensity due to the quenching of the Tb^{3+} by EDTA present in the external buffer. Octyl-*β*-D-glucopyranoside (OG) at 1% w/v final concentrations was used to obtain maximum leakage (100%) that corresponds to minimum fluorescence. The extent of release, R(t), calculated by using the formula $R(t) = 100 \times [I(0) - I(t)]/[I(0) - I(f)]$, where I(0) is the initial fluorescence of the Tb^{3+}:DPA liposomes before addition of membrane-active agents, I(t) is the fluorescence intensity at time t after addition of membrane-active agents, and I(f) is the fluorescence obtained when all the liposome contents leak that corresponds to maximum leakage caused by OG.

REFERENCES

1. Rose SD, *et al*. Functional polarity is introduced by Dicer processing of short substrate RNAs. *Nucleic Acids Res*. 2005; 33 (13): 4140-4156.
2. Afonin KA, *et al*. Co-transcriptional production of RNA-DNA hybrids for simultaneous release of multiple split functionalities. *Nucleic Acids Res*. 2014; 42 (3): 2085-2097.
3. Afonin KA, *et al*. Activation of different split functionalities on re-association of RNA-DNA hybrids. *Nat Nanotechnol*. 2013; 8 (4): 296-304.
4. Afonin KA, *et al*. Design and self-assembly of siRNA-functionalized RNA nanoparticles for use in automated nanomedicine. *Nature Protocols*. 2011; 6 (12): 2022-2034.
5. Afonin KA, *et al*. Triggering of RNA interference with RNA-RNA, RNA-DNA, and DNA-RNA nanoparticles. *ACS Nano*. 2015; 9 (1): 251-259.
6. Afonin KA, *et al*. Multifunctional RNA nanoparticles. *Nano Lett*. 2014; 14 (10): 5662-5671.
7. Nagy KJ, *et al*. Enhanced Mechanical Rigidity of Hydrogels Formed from Enantiomeric Peptide Assemblies. *Journal of the American Chemical Society*. 2011; 133 (38): 14975-14977.
8. Kim T, *et al*. In Silico, *In Vitro*, and *In Vivo* Studies Indicate the Potential Use of Bolaamphiphiles for Therapeutic siRNAs Delivery. *Molecular Therapy-Nucleic Acids*. 2013; 2.
9. Gupta K, *et al*. Mechanism of Membrane Permeation Induced by Synthetic beta-Hairpin Peptides. *Biophysical Journal,* 2013; 105 (9): 2093-2103.
